# Supplementary material for: Sticky collisions of ultracold RbCs molecules
Source: Nat Commun. 2019 Jul 15;10:3104. doi: 10.1038/s41467-019-11033-y (PMC6629645; doi:10.1038/s41467-019-11033-y)
Supplement: Supplementary file 1 — Supplementary Materials [file 41467_2019_11033_MOESM1_ESM.pdf]

Sticky collisions of ultracold RbCs molecules
Supplementary materials

Philip D. Gregory,[1] Matthew D. Frye,[2] Jacob A. Blackmore,[1] Elizabeth M. Bridge,[1] Rahul Sawant,[1] Jeremy M. Hutson,[2,*] and Simon L. Cornish[1,†]

[1]*Joint Quantum Centre (JQC), Durham - Newcastle, Department of Physics, Durham University, Durham DH1 3LE, United Kingdom*

[2]*Joint Quantum Centre (JQC), Durham - Newcastle, Department of Chemistry, Durham University, Durham DH1 3LE, United Kingdom*

I. SUPPLEMENTARY NOTE 1: DERIVATION OF RATE EQUATIONS (2)

We model the rate of change of density $\dot{n}(\mathbf{r},t) = -k_\gamma n(\mathbf{r},t)^\gamma$. Here the power of the density $\gamma = 1,2,3$ corresponds to losses governed by one-, two-, and three-body processes respectively, and k_γ is the collision rate coefficient for the γ-body loss. For a thermal ensemble trapped in a harmonic trap the density is given by

$$n(\mathbf{r},t) = \frac{N_{\rm mol}(t)\omega_x\omega_y\omega_z m^{3/2}}{(2\pi k_{\rm B}T(t))^{3/2}} e^{-\frac{m[\omega_x^2x^2+\omega_y^2y^2+\omega_z^2z^2]}{2k_{\rm B}T(t)}}. \quad (1)$$

Here, $N_{\rm mol}(t)$ is the number of molecules remaining, $T(t)$ is the temperature of the remaining distribution, m is the mass of the molecule, $k_{\rm B}$ is the Boltzmann constant, and ω_x, ω_y, ω_z are the trapping frequencies in the x, y, z directions respectively. The rate of change of the number of molecules can be then obtained by integrating $\dot{n}(\mathbf{r},t)$,

$$\dot{N}_{\rm mol}(t) = \int \dot{n}(\mathbf{r},t) d^3\mathbf{r} = -k_\gamma C^{(\gamma-1)} \left(\frac{N_{\rm mol}(t)^\gamma}{\gamma^{3/2}T(t)^{(3/2)(\gamma-1)}} \right), \quad (2)$$

where $C = (m\bar{\omega}^2/2\pi k_{\rm B})^{3/2}$ and $\bar{\omega} = \sqrt{\omega_x\omega_y\omega_z}$ is the geometric mean of the trapping frequencies.

The temperature of the remaining molecules will change as a function of time. This is due to molecules being preferentially lost from the centre of the trap where the density is highest. The probability that loss occurs at a given location and time can be calculated as $p_{\rm loss}(\mathbf{r}) = \frac{n(\mathbf{r},t)^\gamma}{\int n(\mathbf{r},t)^\gamma d^3\mathbf{r}}$. The total energy of colliding molecules averaged over all molecules can therefore be calculated as

$$E_{\rm avg} = \int p_{\rm loss}(\mathbf{r}) \frac{m}{2} \left(\omega_x^2x^2 + \omega_y^2y^2 + \omega_z^2z^2\right) \, dx \; dy \; dz + \frac{3}{2}k_{\rm B}T = \frac{3k_{\rm B}T(1+\gamma)}{2\gamma}, \quad (3)$$

where the first and second terms in the middle expression are the potential and kinetic energy contributions respectively. The total energy at time t is $3k_{\rm B}T(t)N_{\rm mol}(t)$. If $\Delta N_{\rm mol} = N_{\rm mol}(t) - N_{\rm mol}(t+\delta t)$ molecules are lost in time δt and the temperature changes to $T(t+\delta t)$, the total energy at time $t+\delta t$ is, $3k_{\rm B}T(t+\delta t)[N_{\rm mol}(t) - \Delta N_{\rm mol}] = 3k_{\rm B}T(t)N_{\rm mol}(t) - \Delta N_{\rm mol}E_{\rm avg}$. For small δt this gives a rate equation for the temperature,

$$\dot{T}(t) = \frac{\dot{N}_{\rm mol}(t)}{N_{\rm mol}(t)} \left(\frac{E_{\rm avg}}{3k_{\rm B}} - T(t) \right) = k_\gamma C^{(\gamma-1)} \left(\frac{\gamma-1}{2\gamma} \right) \left(\frac{N_{\rm mol}(t)^{\gamma-1}}{\gamma^{3/2}T(t)^{(3\gamma-5)/2}} \right). \quad (4)$$

We note that for $\gamma = 1$, which corresponds to one-body loss, the temperature is unchanged.

In deriving Supplementary Eq. 4 we have assumed that the molecules remain in thermal equilibrium. Using the single-channel QDT model, we can calculate elastic cross sections as well as loss rates. For our best-fit parameters, the elastic cross section is larger than the loss cross section by a factor of 1.8 at $E = 1.5$ μK×$k_{\rm B}$. In addition, the timescale of the loss is significantly greater than a quarter of the trapping period for all measurements performed. The molecules will therefore thermalize on a timescale comparable to the loss.

We have also assumed that k_γ is constant over the course of a single loss measurement. However, due to the density dependence of the loss, we expect that the temperature of the sample will increase over the course of each measurement.

* j.m.hutson@durham.ac.uk
† s.l.cornish@durham.ac.uk

When fitting the model to the time dependence of the molecule number, we also extract a best estimate for how the sample temperature varies (see Supplementary Fig. 1). The variation in temperature over a single measurement is typically ~ 1 μK. As we have no method of experimentally verifying this temperature change, we use the starting temperature in our analysis. For our best-fit parameters, shown in Fig. 3, the loss rate is relatively flat as a function of temperature, certainly below 2.5 μK, and we therefore do not expect this assumption to have much impact. The stronger dependence of k_2 on collision energy at higher temperatures may explain the slight deviation between theory and experiment for the highest-temperature investigated.

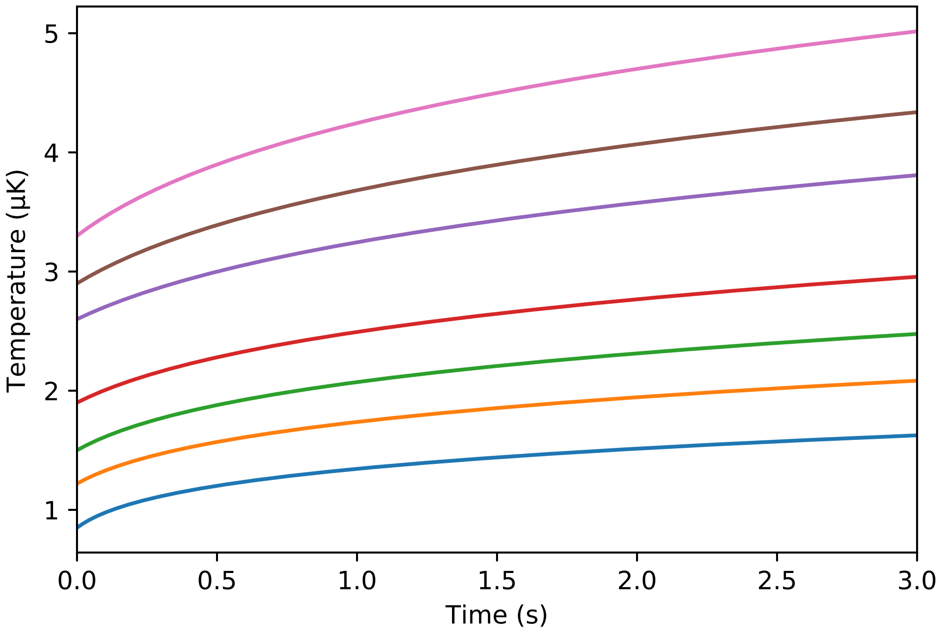

SUPPLEMENTARY FIG. 1. **Time evolution of the sample temperature**. Temperature according to the fitted model assuming fixed k_2 for each experimental measurement in Fig. 3 in the main text.

II. SUPPLEMENTARY NOTE 2: EXPERIMENTS AT DIFFERENT MAGNETIC FIELDS

As discussed in the main text, to investigate loss of molecules at a range of magnetic fields, we transfer the molecules to the ground state with $B = 181.5$ G. We then ramp the magnetic field to the desired value linearly over 50 ms. After a variable hold time, B is ramped back to 181.5 G over a further 50 ms before dissociation and imaging. The measured loss rates for a range of magnetic fields between 4.6 G and 229.8 G are shown in Supplementary Fig. 2.

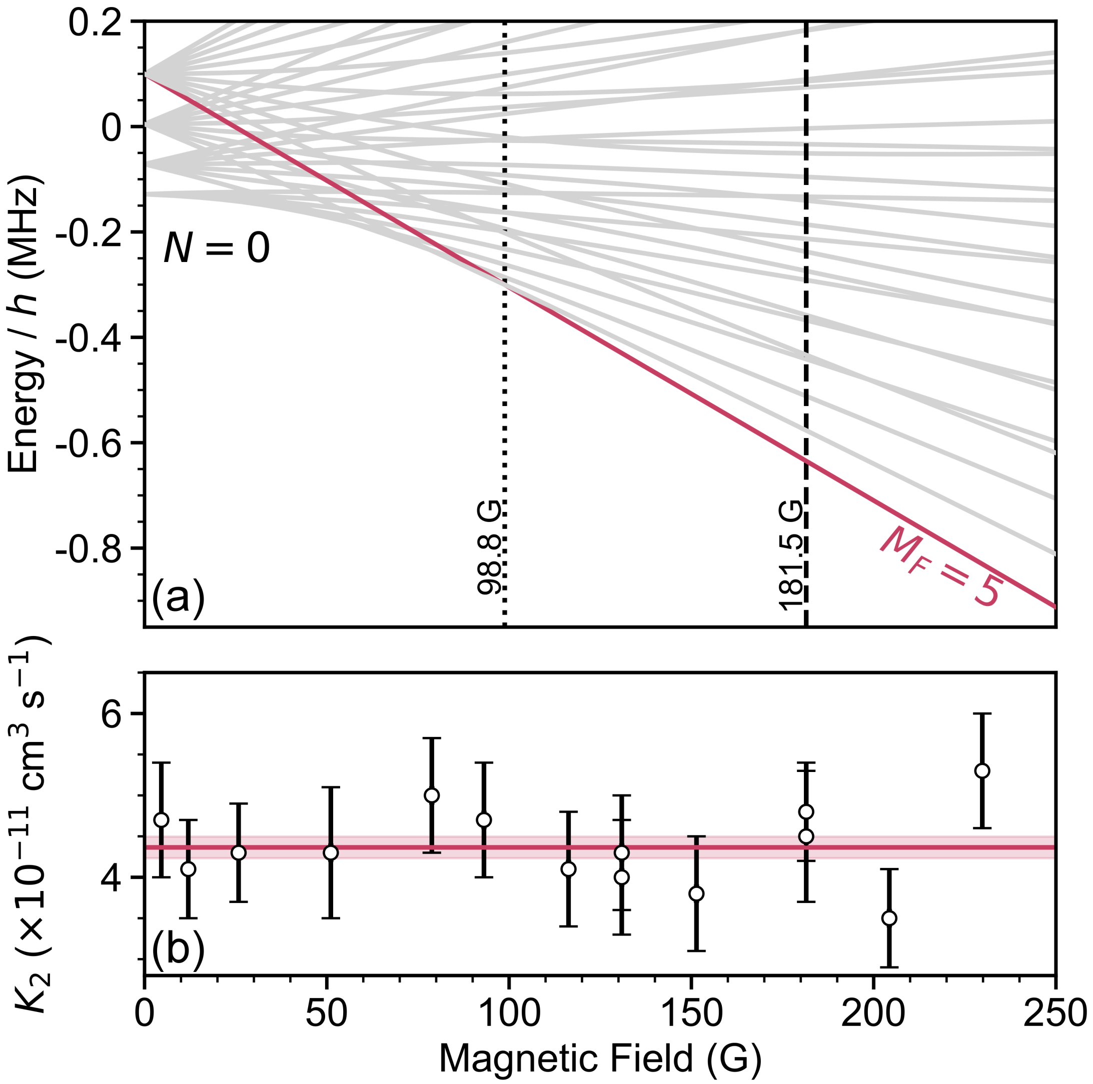

SUPPLEMENTARY FIG. 2. **Dependence of loss rate on magnetic field.** (a) Hyperfine Zeeman structure of RbCs in the rotational ground state. Molecules occupy the spin-stretched state $N = 0, M_F = +5$ highlighted. (b) Measured two-body loss rate coefficient as a function of magnetic field for molecules with initial temperature $T = 1.5$ μK. The error bars show the $1-\sigma$ uncertainty in the measured loss rate coefficient k_2. We observe no variation in the loss rate even when the occupied hyperfine state is no longer the lowest energy. The horizontal red line indicates the average loss rate coefficient of 4.4(1) $\mathrm{cm}^3\ \mathrm{s}^{-1}$ across all the results shown.

III. SUPPLEMENTARY NOTE 3: NUMERICAL VALUES FOR RESULTS SHOWN IN FIG. (4) IN THE MAIN TEXT

(N, M_F)	E/h (kHz)	$k_2^{\mathrm{univ}}(0)$ ($\times 10^{-11}$ cm^3 s^{-1})	$k_2^{\mathrm{univ}}(1.5\ \mu\mathrm{K})$ ($\times 10^{-11}$ cm^3 s^{-1})	$k_2^{\mathrm{exp.}}$ ($\times 10^{-11}$ cm^3 s^{-1})
(0, +5)*	0	17.9	9.9	4.8(6)
(0, +5)*	0	17.9	9.9	4.5(8)
(0, +4)	58	17.9	9.9	6.3(7)
(0, +4)*	201	17.9	9.9	5.8(5)
(0, +4)	201	17.9	9.9	6.4(7)
(1, +5)	980,231	17.9	9.9	6.4(9)
(1, +6)	980,385	16.3	9.4	6.2(8)
(2, +7)	2,941,090	15.8	9.1	9(1)

SUPPLEMENTARY TABLE I. **Loss rates measured in a range of rotational and hyperfine states.** Molecules are prepared in a state (N, M_F) with $T = 1.5$ μK and $B = 181.5$ G. The energy E of each state is given with respect to the state (0, +5), which at this magnetic field has the lowest energy. Asterisks indicate measurements where the state is populated directly with STIRAP.

IV. SUPPLEMENTARY NOTE 4: THERMALLY AVERAGED LOSS RATE COEFFICIENTS AT 8.7 μK

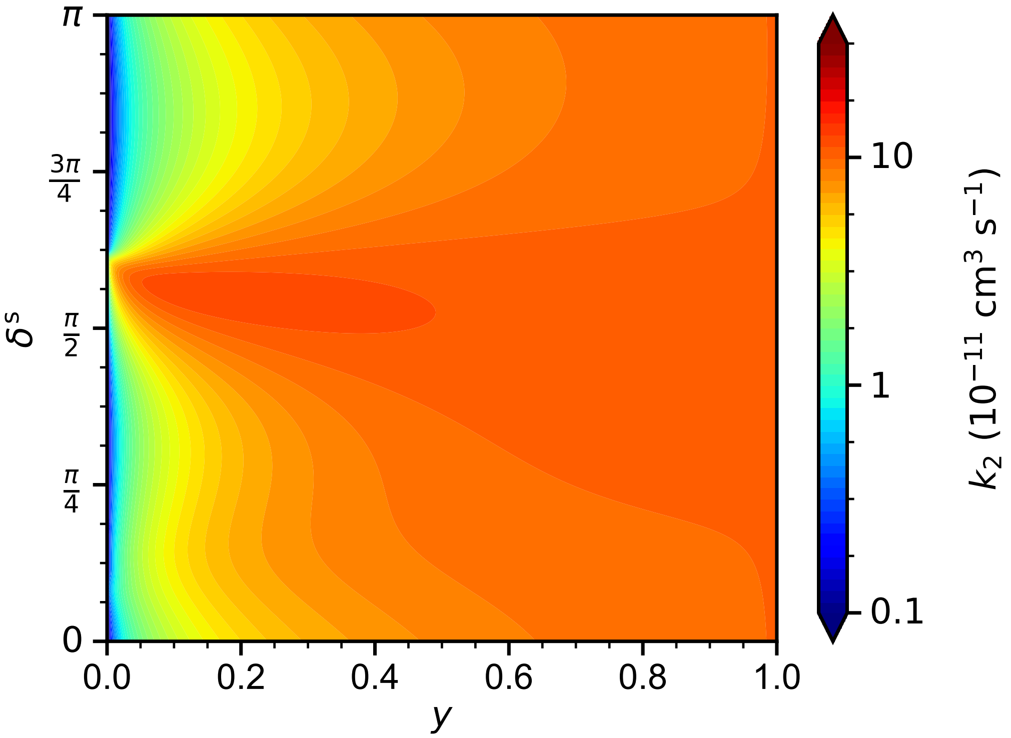

SUPPLEMENTARY FIG. 3. **Contour plot of thermally averaged loss rate coefficients k_2 for RbCs from the single-channel model at 8.7 μK**. This temperature corresponds to measurements of loss reported in [1]. In this work, Takekoshi *et al*. observed fast loss with rates up to 10^{-9} cm^3 s^{-1} in magnetic fields below $\sim$ 90 G, which they attributed to hyperfine-changing collisions. However, loss rates this high are larger than the maximum allowed by reflection off the long-range potential at the temperature of the experiment.

[1] Takekoshi, T. *et al.* Ultracold dense samples of dipolar RbCs molecules in the rovibrational and hyperfine ground state. *Phys. Rev. Lett.* **113**, 205301 (2014).
